# Expression of histone deacetylase (HDAC) family members in bortezomib-refractory multiple myeloma and modulation by panobinostat

**DOI:** 10.20517/cdr.2021.44

**Published:** 2021-09-23

**Authors:** Tiewei Cheng, Kendall Kiser, Leslie Grasse, Lakesla Iles, Geoffrey Bartholomeusz, Felipe Samaniego, Robert Z. Orlowski, Joya Chandra

**Affiliations:** ^1^Department of Pediatrics Research, University of Texas MD Anderson Cancer Center, Houston, TX 77030, USA.; ^2^Department of Experimental Therapeutics, University of Texas MD Anderson Cancer Center, Houston, TX 77030, USA.; ^3^Department of Lymphoma and Myeloma, University of Texas MD Anderson Cancer Center, Houston, TX 77030, USA.; ^4^McGovern Medical School, University of Texas Health Science Center at Houston, Houston, TX 77030, USA.; ^5^Department of Epigenetics and Molecular Carcinogenesis, University of Texas MD Anderson Cancer Center, Houston, TX 77030, USA.

**Keywords:** Histone deacetylase, bortezomib resistance, selective HDAC inhibitors, HDAC6, HDAC7

## Abstract

**Aim:** Multiple myeloma (MM) is a hematological malignancy of antibody-producing mature B cells or plasma cells. The proteasome inhibitor, bortezomib, was the first-in-class compound to be FDA approved for MM and is frequently utilized in induction therapy. However, bortezomib refractory disease is a major clinical concern, and the efficacy of the pan-histone deacetylase inhibitor (HDACi), panobinostat, in bortezomib refractory disease indicates that HDAC targeting is a viable strategy. Here, we utilized isogenic bortezomib resistant models to profile HDAC expression and define baseline and HDACi-induced expression patterns of individual HDAC family members in sensitive *vs.* resistant cells to better understanding the potential for targeting these enzymes.

**Methods:** Gene expression of HDAC family members in two sets of isogenic bortezomib sensitive or resistant myeloma cell lines was examined. These cell lines were subsequently treated with HDAC inhibitors: panobinostat or vorinostat, and HDAC expression was evaluated. CRISPR/Cas9 knockdown and pharmacological inhibition of specific HDAC family members were conducted.

**Results:** Interestingly, HDAC6 and HDAC7 were significantly upregulated and downregulated, respectively, in bortezomib-resistant cells. Panobinostat was effective at inducing cell death in these lines and modulated HDAC expression in cell lines and patient samples. Knockdown of HDAC7 inhibited cell growth while pharmacologically inhibiting HDAC6 augmented cell death by panobinostat.

**Conclusion: **Our data revealed heterogeneous expression of individual HDACs in bortezomib sensitive *vs.* resistant isogenic cell lines and patient samples treated with panobinostat. Cumulatively our findings highlight distinct roles for HDAC6 and HDAC7 in regulating cell death in the context of bortezomib resistance.

## INTRODUCTION

Multiple myeloma is an incurable clonal B-cell malignancy characterized by the accumulation of terminally differentiated, antibody-producing plasma cells in the bone marrow^[1]^. Bortezomib, a proteasome inhibitor, is a cornerstone of anti-myeloma therapy and is frequently used as a component of frontline therapy^[2-5]^. However, acquired or secondary resistance consistently emerges in patients who initially respond to bortezomib or other proteasome inhibitor-based therapies^[6]^ suggesting a role for epigenetic alterations as contributors to resistance^[7]^. This concept was validated by the clinical efficacy of the histone deacetylase inhibitor (HDACi), panobinostat, which was approved by the FDA for the treatment of refractory and relapsed multiple myeloma^[8-11]^. Broadly, HDACi represent a group of epigenetic agents that modify gene expression to restore normal differentiation and programmed cell death^[12]^ and have emerged as a novel class of anti-cancer drugs, especially in leukemia, lymphoma, and myeloma^[13]^. HDACi regulate histone acetylation, a key epigenetic mark that influences gene expression, which is coordinately controlled by families of enzymes that are histone deacetylases (HDACs) and histone acetyltransferases, which in turn regulate various key cellular functions such as gene transcription, cell differentiation, cell cycle progression, and apoptosis^[14]^.

Panobinostat is a pan-HDACi with the greatest potency against class I, II, and IV histone deacetylases. Class I (HDAC1, 2, 3, and 8), class II (HDAC4, 5, 6, 7, 9, and 10), and class IV (HDAC11) are zinc-dependent amidohydrolases that share the presence of a conserved deacetylase domain. Class II HDACs are further subdivided into class IIa (HDAC4, 5, 7, and 9) and class IIb (HDAC6 and 10). HDAC6, a class IIb HDAC subfamily member, is unique among family members by having two active catalytic domains and is expressed primarily in the cytoplasm^[15]^. HDAC6 is known to target several non-histone substrates like alpha-tubulin and HSP90 that control critical cellular functions, including protein degradation, trafficking, and folding^[16]^. HDACi with selectivity for HDAC6 has been developed and explored as single agents or components of combination therapy strategies for multiple myeloma^[17]^. HDAC7, a member of the HDAC IIa subfamily^[18]^, differs from other HDACs because it has a long N terminal domain that enables HDAC7 to bind transcription factors and facilitates export to the cytoplasmic compartment in addition to its expected and conventional histone acetylation removal function^[19,20]^. The N terminal domain has adaptor and phosphorylation domains in addition to the conventional C terminal catalytic domain. Decreased HDAC7 priming for cell death has been observed in neurons exposed to low potassium treatment, but not documented in myeloma^[21]^. Interestingly both HDAC6 and 7 exert immune-modulatory effects in myeloma^[22,23]^ but have not been specifically studied in the context of bortezomib refractory disease.

The toxicity profile of panobinostat, which leads to poor tolerability, frequently limits its usage in combination with other commonly used agents treating multiple myeloma^[24,25]^; therefore, dosing/combination regimens that enable optimization of panobinostat’s efficacy without severe toxicities is clinically relevant. Recently the pivotal PANORAMA 3, an open-label, randomized, phase 2 study, demonstrated improved tolerability, particularly far less GI toxicity, by combining oral panobinostat with subcutaneous bortezomib plus oral dexamethasone while achieving similar efficacy as intravenously delivered bortezomib^[8,26]^. However, panobinostat’s specific molecular mechanism of action is still largely unknown given its broad inhibitory effects on HDACs^[27]^, which leaves the door open to further refine the strategy of targeting specific HDAC family members. This may further improve the toxicity profile of targeting HDACs while retaining anti-myeloma efficacy. This may be particularly true of class I HDACs, which have been shown to be targets of bortezomib-induced cytotoxicity^[28]^. However, very few studies have examined specific HDAC family members in the context of bortezomib-containing regimens. Work by Kikuchi *et al.*^[28]^ reports that bortezomib causes downregulation of HDAC1, 2, and 3 at the transcriptional level and that overexpression of HDAC1 conferred resistance to bortezomib; however, HDAC expression in bortezomib-refractory models was not probed.

Here, we specifically examine bortezomib-sensitive and bortezomib-resistant isogenic myeloma cell lines to understand the potential for targeting specific HDAC family members and how HDAC expression is regulated by panobinostat exposure. We find heterogeneous expression of HDAC family members in bortezomib-sensitive and resistant lines. Interestingly, panobinostat treatment altered HDAC gene expression: HDAC7 was downregulated while HDAC6 was upregulated after panobinostat treatment in myeloma cell lines. This was also observed in patients treated with panobinostat. Knockdown of HDAC7 partially recapitulated panobinostat-induced cell death, and panobinostat synergized with HDAC6 selective inhibitors to induce cell death, highlighting potential strategies for more focused targeting of HDAC family members in bortezomib-refractory disease.

## METHODS

### Tissue culture and reagents

Multiple myeloma and lymphoma cell lines RPMI-8226, Kas6, Daudi, and SUDHL4, were from American Type Culture Collection (ATCC, Manassas, VA). To generate bortezomib-refractory cells, parental RPMI-8226 or Kas6 cells (designated as RMPI-8226wt or Kas6wt) were chronically exposed to increasing concentrations of bortezomib to generate resistant lines (designated as RMPI-8226v10r or Kas6v10r)^[29,30]^. Resistant lines were then cultured in the presence of 10 nM bortezomib. Cell line identities were confirmed by Short Tandem Repeat DNA profiling as conducted by the MD Anderson Cancer Center Characterized Cell Line Core. All cell lines were cultured in RPMI1640 (Thermo Fisher Scientific, Waltham, MA) with 10% FBS (Thermo Fisher Scientific, Waltham, MA), 1% L-glutamine (Thermo Fisher Scientific, Waltham, MA), and 1% penicillin/streptomycin (Lonza, Basel, Switzerland) at 37 °C in a 5% CO_2_ incubator. All cultures were free of bacterial, fungal, and mycoplasma contamination (mycoplasma contamination was tested every 6 months). The following drugs were used for the study: bortezomib, LC Laboratories, (Woburn, MA); vorinostat, Cayman Chemical, (Ann Arbor, MI); panobinostat, LC Laboratories, (Woburn, MA); ricolinostat, Selleck Chemicals, (Houston, TX); tubacin, Selleck Chemicals, (Houston, TX).

### Real-time qPCR

Total RNA was isolated using RNeasy Mini Kit (QIAGEN, Hilden, Germany) from 3 million cells. Reverse transcription reaction was performed for each sample (1 μg of RNA) via iScript RT kit (Bio-Rad, Hercules, CA) per the manufacturer’s protocol. Real-time qPCR was carried out using the iTaq Universal SYBR Green PCR master mix (Bio-Rad, Hercules, CA) in a 20 μL total volume. The PCR conditions were described previously^[19]^. The actin gene was used as an internal control to normalize the amount of amplifiable RNA. The comparative CT method was used to determine relative gene expression for each target gene. Primers were synthesized by Sigma-Aldrich (Sigma-Aldrich, St. Louis, MO), and sequences are listed in Table 1.

**Table 1:** Primers use for RT-qPCR for HDAC family members

Genes	Forward (5’ -> 3’)	Reverse (5’ -> 3’)
*HDAC1*	CTACTACGACGGGGATGTTGG	GAGTCATGCGGATTCGGTGAG
*HDAC2*	ATGGCGTACAGTCAAGGAGG	TGCGGATTCTATGAGGCTTCA
*HDAC3*	CCTGGCATTGACCCATAGCC	CTCTTGGTGAAGCCTTGCATA
*HDAC4*	GGCCCACCGGAATCTGAAC	GAACTCTGGTCAAGGGAACTG
*HDAC5*	TCTTGTCGAAGTCAAAGGAGC	GAGGGGAACTCTGGTCCAAAG
*HDAC6*	AAGAAGACCTAATCGTGGGACT	GCTGTGAACCAACATCAGCTC
*HDAC7*	GGCGGCCCTAGAAAGAACAG	CTTGGGCTTATAGCGCAGCTT
*HDAC8*	TCGCTGGTCCCGGTTTATATC	TACTGGCCCGTTTGGGGAT
*HDAC9*	AGTAGAGAGGCATCGCAGAGA	GGAGTGTCTTTCGTTGCTGAT
*HDAC10*	CAGTTCGACGCCATCTACTTC	CAAGCCCATTTTGCACAGCTC
*HDAC11*	ACCCAGACAGGAGGAACCATA	TGATGTCCGCATAGGCACAG
Actin	AAGGAGCCCCACGAGAAAAAT	ACCGAACTTGCATTGATTCCAG

### *In vitro* viability assay and DNA fragmentation

Cells (2 × 10^5^) were plated in 12-well plates 24 h before drug treatment. After drug incubation, cell viability was measured by trypan blue exclusion assay using a Vi-CELL® (Beckman Coulter, Inc., Pasadena, CA). For analysis of DNA fragmentation, cells were harvested and pelleted by centrifugation. The pellets were then re-suspended in PBS containing 50 μg/mL propidium iodide (PI) (Sigma, St. Louis, MO), 0.1% Triton X-100 (Thermo Fisher Scientific, Waltham, MA), and 0.1% sodium citrate (Sigma, St. Louis, MO). DNA PI staining was measured by fluorescence-activated cell-sorting analysis using the FL-3 channel (FACSCalibur flow cytometer; Becton Dickinson). Cells displaying a hypodiploid DNA content that is indicative of DNA fragmentation were scored as apoptotic.

### Immunoblotting

Cells were harvested and lysed in Triton lysis buffer containing PBS (GE Healthcare, Chicago, IL) with 1% Triton X-100 (Thermo Fisher Scientific, Waltham, MA), 25 mM Tris (Thermo Fisher Scientific, Waltham, MA), pH 7.5, 150 mM NaCl (Thermo Fisher Scientific, Waltham, MA), and a protease inhibitor cocktail (Roche, Mannheim, Germany) for 1 h at 4 °C. Sample protein concentration was measured by Bradford assay (Bio-Rad Laboratories, Hercules, CA). Lysates were boiled in a sample buffer containing 62.5 mmol/L Tris-HCl (pH 6.8; Tris, Thermo Fisher Scientific, Waltham, MA; HCl, Thermo Fisher Scientific, Waltham, MA), 10% (w/v) glycerol (Thermo Fisher Scientific, Waltham, MA), 100 mmol/L DTT (Sigma, St. Louis, MO), 2.3% SDS (Thermo Fisher Scientific, Waltham, MA), and 0.002% bromophenol blue (Thermo Fisher Scientific, Waltham, MA) for 5 min and cooled on ice for additional 5 min. Lysates were then subjected to SDS-PAGE at 110V for 1-1.5 h and subsequently transferred to PVDF membrane (Bio-Rad, Hercules, CA) at 100V for 1 h. The membranes were incubated in TBS blocking buffer containing 10 mmol/L Tris-HCl (pH 8.0), 150 mmol/L NaCl (Thermo Fisher Scientific, Waltham, MA), and 5% nonfat milk (Bio-Rad, Hercules, CA) for 1 h at room temperature while shaking, and then rinsed once briefly with TBS-T containing 0.1% Tween-20 (Thermo Fisher Scientific, Waltham, MA). The membranes were subsequently incubated with primary antibodies (HDAC6, Cell Signaling, Danvers, MA; HDAC7, Abcam, Cambridge, United Kingdom; Actin, Sigma, St. Louis, MO) diluted 1:1000 in blocking buffer overnight 4 °C, followed by TBS-T washing, and incubation with secondary antibodies (anti-mouse HRP, GE Healthcare, Chicago, IL; anti-rabbit HRP, GE Healthcare, Chicago, IL) diluted 1:8000-1:10,000 in blocking buffer for 1 h at room temperature while shaking. Proteins were visualized with ECL (Bio-Rad, Hercules, CA), and densitometry was measured with NIH ImageJ (Bethesda, MA).

### Gene knockout

To knock out HDAC7 gene expression in RPMI-8226wt cells, we designed six guide RNAs (gRNAs) specific to a sequence in the HDAC7 region. The RMPI-8226wt cells were transiently electroporated with 240 ng of each gRNA mixed with 500 ng Invitrogen GeneArt™ Platinum™ Cas9 Nuclease (Invitrogen Carlsbad, CA) using the Invitrogen Neon Transfection System (Invitrogen, Carlsbad, CA) at 1100 v, 30 ms, and 2 pulses. Subsequently, protein lysates harvested from cells with each of the six gRNAs were immunoblotted for HDAC7 to identify the gRNA that provided the most decrease of *HDAC7* gene expression (5’-AAACCCCCTGGATGCACAGCCCCGGCG-3’) among the 6 gRNA. Afterward, cells with the above-mentioned gRNA were sorted as single cells in CoSTAR ultra-low cluster, 96-well plates (Corning Inc, Corning, NY), with conditioned media to allow single-cell cloning and expansion. This process was repeated once to identify sub-clones #1 and #2 with sufficient knockdown of *HDAC7* gene expression.

### Statistical analysis and combination index

Statistical analysis was performed using GraphPad Prism Software (GraphPad, San Diego, CA). As appropriate, raw data or percentage was compared by unpaired Student’s *t*-test or Mann Whitney test. Values were given as the mean ± SEM of triplicate or more. Statistical significance was set at *P* < 0.05. Synergy was calculated using isobologram analysis based on the Chou and Talalay method by Calcusyn software^[20]^ (Biosoft, Ferguson, MO), with combination index (CI) values < 1 considered synergistic.

## RESULTS

### Differential expression of HDACs in isogenic bortezomib-resistant and sensitive myeloma cells

**Figure 1:**
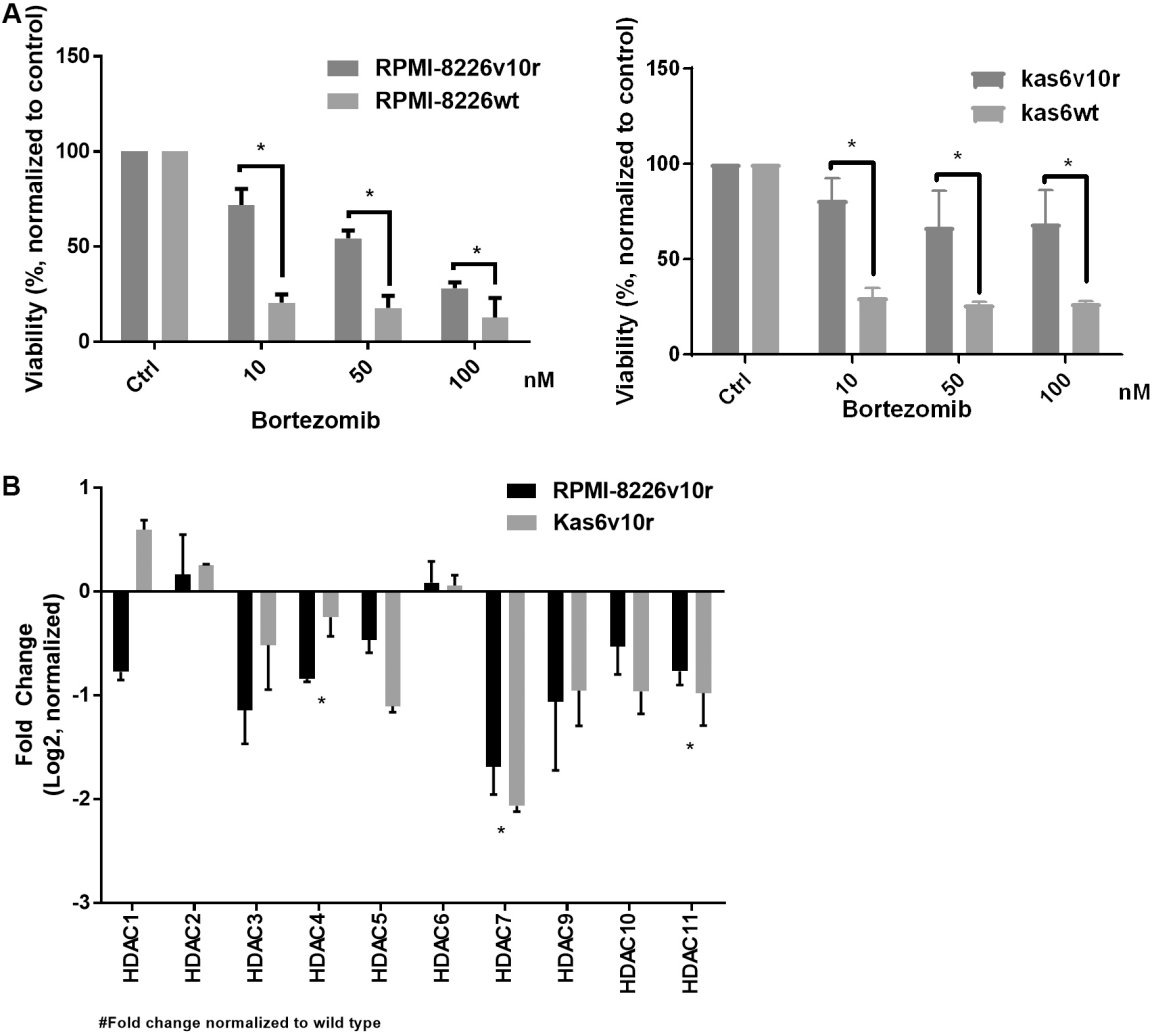
Resistance to bortezomib results in heterogeneous expression of histone deacetylases (HDACs). (A) Myeloma cells (RMPI-8226wt and Kas6wt) and resistant counterparts (RPMI-8226v10r and Kas6v10r) were treated with various concentrations of bortezomib (10, 50, 100 nM) for 24 h, and cell viability was measured. Viability was normalized to control treated with vehicle. (B) Expression of HDACs in myeloma cells (RMPI-8226 and Kas6) was measured by RT-PCR. Individual HDAC expression of RPMI-8226v10r and Kas6v10r was normalized to RPMI-8226wt and Kas6wt, respectively. Data was then log2 transformed, and positive numbers indicated increased expression while negative numbers indicated decreased expression in v10r cells. ^*^Indicate significant (*P* < 0.05) changes in viability for (A) or transcript expression for (B).

Previous work has shown that bortezomib regulates the expression of HDAC family members^[28]^ and that HDAC1 overexpression conferred resistance to bortezomib whereas knockdown conferred sensitivity^[28]^. These data prompted us to investigate the status of HDAC family members in models of established bortezomib resistance. Bortezomib-resistant cell lines were generated from two different human myeloma cell lines (RPMI-8226 and Kas6)^[29,30]^. These lines did not show mutations in the B5 subunit of the proteasome, as has been reported in patients with bortezomib refractory disease; however, increased activation of insulin-like growth factor receptor-1 (IGFR-1) due to autocrine and paracrine secretion of IGF1 has been cited as the mechanism of bortezomib resistance in these lines^[19]^. In order to independently confirm the resistance of the lines, we treated cells with increasing doses of bortezomib and found that viability, as measured by trypan blue positivity, was significantly reduced in parental (RPMI-8226wt or Kas6wt) cells but not in resistant (RPMI-8226v10r or Kas6v10r) cells. As shown in Figure 1A, both resistant myeloma cells RPMI-8226v10r and Kas6v10r showed significantly less reduction in viability than their parental counterparts after bortezomib treatment. For example, 24 h of exposure to 10 nM or 50 nM of bortezomib reduced viability to 71.9% or 54.5% in RPMI-8226v10r cells, respectively, whereas the same concentrations of bortezomib-induced a greater loss of viability (20.6% or 17.8%) in RPMI-8226wt cells. Similar observations were made in Kas6v10r *vs.* Kas6wt, with the difference in viability being significant upon exposure to 10, 50, or 100 nM bortezomib [Figure 1A]. The sensitive and resistant lines were then profiled for expression of HDAC family member gene expression. The data demonstrated the consistency of up- or down-regulation of HDACs (with the exception of HDAC1) in the two bortezomib-resistant models, RPMI-8226v10r and Kas6v10r [Figure 1B] when normalized to expression in the sensitive parental cells. Interestingly, we found that alteration of HDAC expression in bortezomib-resistant myeloma cells is heterogeneous, such that HDAC3, 4, 5, 7, 9, 10, and 11 are downregulated while HDAC2 and 6 are upregulated [Figure 1B].

**Figure 2:**
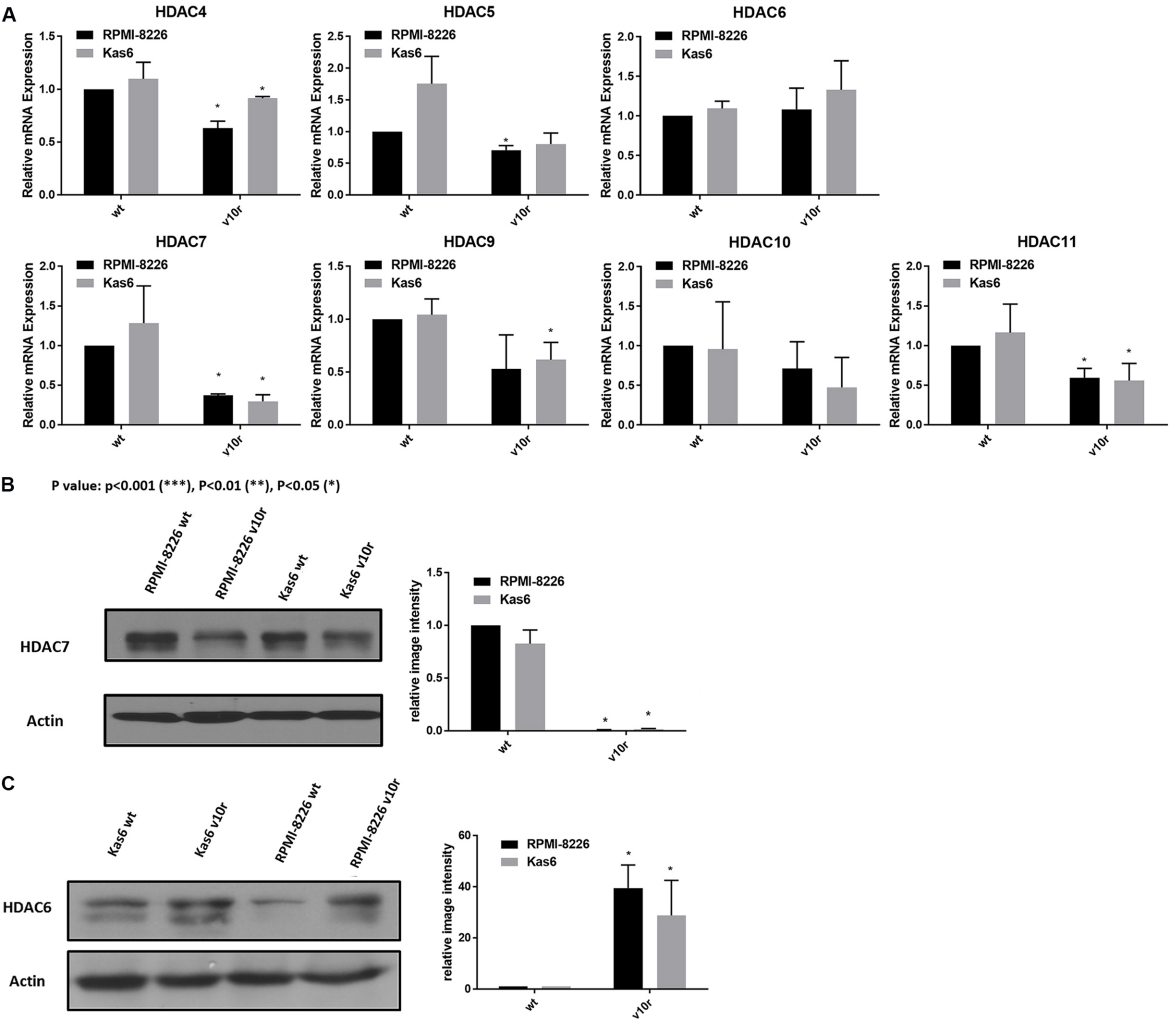
Altered expression of HDAC6 and HDAC7 among class II and IV HDACs in bortezomib resistant cells. (A) Expression of class II and IV HDACs in myeloma cells (RMPI-8226 and Kas6) was measured by RT-PCR. Expression of individual HDAC in RMPI-8226v10r, Kas6wt, and Kas6v10r was normalized to RPMI-8226wt and plotted in separate figures. (B) HDAC6 protein expression was measured using immunoblotting. Results were quantified from three replicates by measuring band intensity using ImageJ. Quantitative results are displayed graphically next to the immunoblotting image from a representative blot. (C) HDAC7 protein expression was measured using immunoblotting. Results were quantified from three replicates by measuring band intensity using ImageJ. Quantitative results are displayed graphically next to the immunoblotting image from a representative blot. HDACs: Histone deacetylases.

To facilitate comparison across the two different models of bortezomib resistance, we examined the expression of class II and IV HDAC family members individually. Both RPMI-8226v10r and Kas6v10r showed HDAC7 as the most down-regulated family member among class II and IV HDACs, compared to parental counterparts. In addition, we found that HDAC6 was upregulated in bortezomib-resistant myeloma cells *vs.* wildtype cells [Figure 2A]; however, the degree of upregulation did not achieve statistical significance. HDAC6 and HDAC7 protein expression was probed to determine if transcript changes were seen at the protein level in bortezomib-resistant myeloma cells. HDAC7 protein expression was significantly downregulated, while HDAC6 protein expression was upregulated in both RPMI8226v10r and Kas6v10r cells compared to wildtype cells [Figure 2B and C].

### Panobinostat but not vorinostat induced significant cell death in resistant myeloma cells, and altered expression of class II and IV HDACs

**Figure 3:**
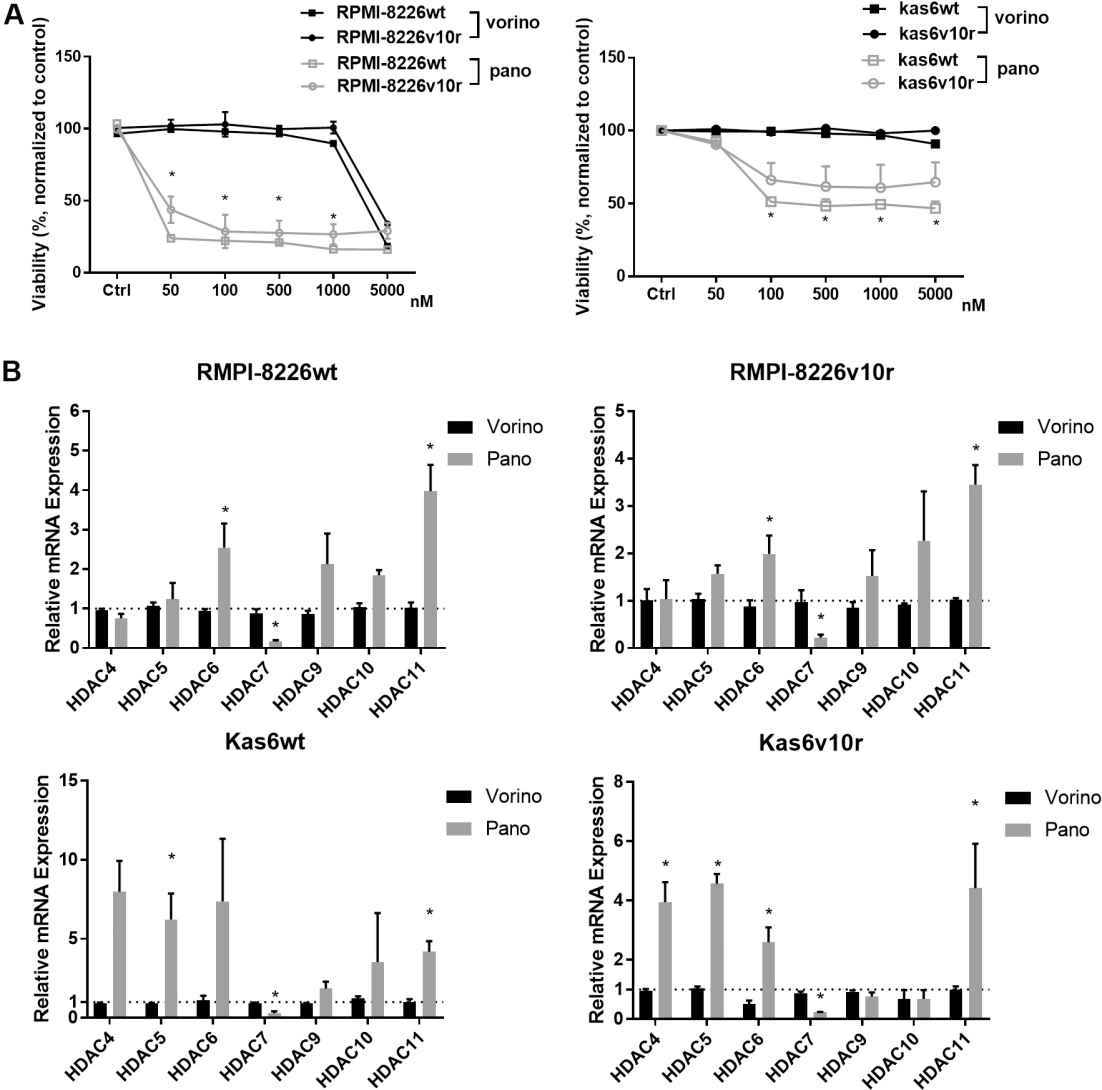
Panobinostat alters expression of class II and IV HDACs. (A) Myeloma cells (RMPI-8226wt or v10r or and Kas6wt or v10r) were treated with various concentrations of vorinostat (50, 100, 500, 1000, and 5000 nM) or panobinostat (50, 100, 500, 1000, and 5000 nM) for 48 h, and cell viability was measured. Viability was normalized to control treated with vehicle. (B) RMPI-8226wt or v10r and Kas6wt or v10r were treated with 50nM vorinostat or panobinostat for 12 h before harvesting for total RNA, and mRNA expression of class II and IV HDACs was measured by RT-PCR. Individual HDAC expression in RMPI-8226 and Kas6 cells treated with vorinostat or panobinostat was normalized to that in respective RMPI-8226 and Kas6 cells treated with vehicle only. ^*^P < 0.05. HDACs: Histone deacetylases.

Given the altered expression of HDAC family members between parental and bortezomib-resistant myeloma cells, we investigated whether pan-HDAC inhibition using vorinostat or panobinostat would induce differential responses. RPMI-8226 and Kas6 myeloma cells were treated with various concentrations of either vorinostat or panobinostat for 48 h. Neither RPMI-8226v10r nor Kas6v10r responded to vorinostat or panobinostat differently compared to wildtype RPMI-8226wt or Kas6wt, respectively [Figure 3A], indicating that bortezomib resistance did not influence sensitivity to HDACi. Interestingly, panobinostat induced substantial cell death in both RPMI-8226 and Kas6 cells regardless of bortezomib-resistance status. In contrast, both RPMI-8226 and Kas6 cells remained resistant to vorinostat at the concentrations tested, which was consistent with literature^[13,14]^ and indicated reduced potency of vorinostat compared to panobinostat. To determine whether this reduced potency correlated with altered expression of class II and IV HDAC, we examined transcript levels after vorinostat and panobinostat treatment. Vorinostat exposure did not change the expression of HDAC4, 5, 6, 7, 9, 10, or 11. However, panobinostat significantly altered class II and IV expression among RPMI-8226 and Kas6 myeloma cells and correlated with the efficacy of panobinostat in these lines [Figure 3B]. The majority of class II and IV HDACs were upregulated after panobinostat treatment, although the level of increase varied between wildtype and bortezomib-resistant RPMI-8226 and Kas6 cells. In contrast to the upregulation of most class II and IV HDACs by panobinostat, HDAC7 expression was unique in that it was consistently downregulated by panobinostat in both RPMI-8226 wildtype and bortezomib-resistant cells as well as Kas6 wildtype and bortezomib-resistant cells [Figure 3B].

### HDAC7 was downregulated by panobinostat in both myeloma cells and patient samples, and knockdown of HDAC7 slowed cell growth

**Figure 4:**
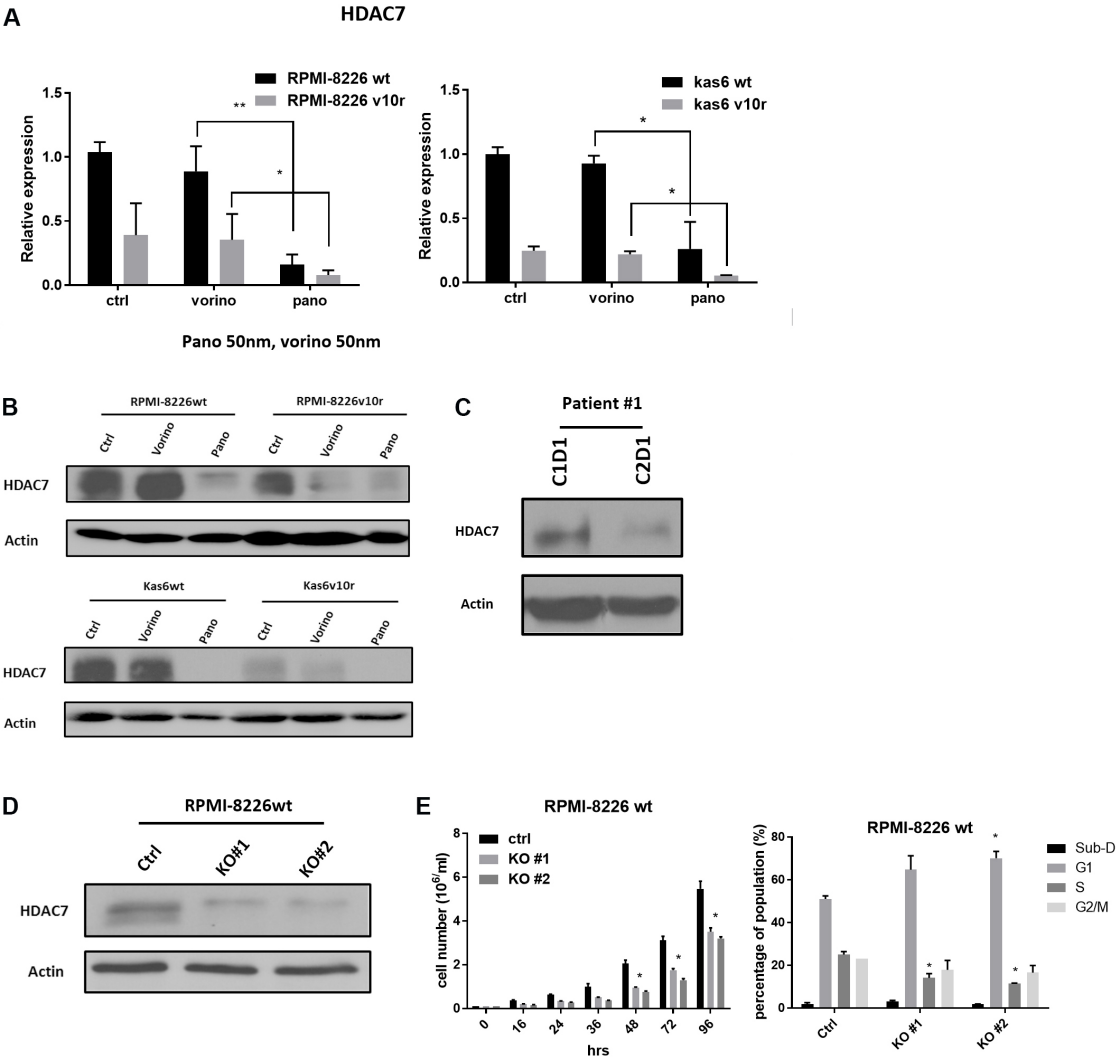
HDAC7 is downregulated by panobinostat and knockdown causes growth inhibition in bortezomib-sensitive and refractory lines. (A) RMPI-8226 and Kas6 were treated with 50 nM vorinostat or panobinostat for 12 h before the harvest of total RNA. HDAC7 expression was measured by RT-PCR. Expression of HDAC7 in sensitive or resistant RMPI-8226 and Kas6 cells treated with vorinostat or panobinostat was normalized to vehicle-treated RPMI-8226wt and Kas6wt, respectively. (B) RMPI-8226 and Kas6 were treated with 50 nM vorinostat or panobinostat for 12 h before harvesting for protein, and HDAC7 expression was measured by immunoblotting. (C) Peripheral blood mononuclear cells from a patient enrolled from a phase I/II newly diagnosed multiple myeloma clinical trial (Clinicaltrials.gov, number NCT01440582) before and after the first cycle treatment was harvested, and HDAC7 expression in PBMC was measured by immunoblotting. (D, E) RPMI-8226wt transfected with either vehicle or cas9 plasmid with sgRNA targeting HDAC7. HDAC7 after transfection was measured by immunoblotting. Cell growth was monitored by measuring the number of viable cells over the course of 96 h in (D). In addition, cell cycle distribution was determined by propidium iodide staining using flow cytometer in (E). ^*^P < 0.05. ^**^P < 0.01. HDAC: Histone deacetylase.

We further examined HDAC7 expression before and after vorinostat or panobinostat treatment in wildtype and bortezomib-resistant myeloma cells individually. Figure 4A shows that resistant cells had decreased expression of HDAC7 and that a further significant reduction in HDAC7 expression was seen after exposure to panobinostat in resistant RPMI-8226 and in Kas6 cells. This was not seen in vorinostat treated resistant cells, which did not show a difference in HDAC7 expression compared to vehicle-treated control cells. Immunoblotting confirmed the decrease of HDAC7 expression by panobinostat in both RPMI-8226 and Kas6 cells [Figure 4B]. To further corroborate the observation, we obtained peripheral blood samples from a newly diagnosed multiple myeloma patient who was treated in a clinical trial regimen containing panobinostat at the University of Texas MD Anderson Cancer Center. Measurement of HDAC7 protein expression pre-panobinostat (cycle 1 day 1) and post panobinostat (cycle 2 day 1) showed a decrease of more than 50% after treatment [Figure 4C]. Given the correlation between increased cell death and decreased HDAC7 expression by panobinostat, we hypothesized that downregulation of HDAC7 was linked to the reduced cell viability induced by panobinostat. To test this hypothesis, HDAC7 was knocked-out using the CRISPR/Cas9 system in RPMI-8226 parental cells. The decrease of HDAC7 expression was confirmed by immunoblotting [Figure 4D]. Cell growth was significantly slowed in RPMI-8226wt cells with HDAC7 knockdown compared to control cells [Figure 4E]. Further analysis of cell cycle distribution revealed an increase in the G1 population and a decrease in the S phase population compared to the control cells in which HDAC7 was not reduced [Figure 4E]. Together, these data indicate that reducing expression of HDAC7 in parental myeloma cells, which is a feature of panobinostat treatment, caused cell cycle alterations likely responsible for the observed reduction in cell growth.

### Panobinostat causes upregulation of HDAC6 in myeloma and lymphoma cells

**Figure 5:**
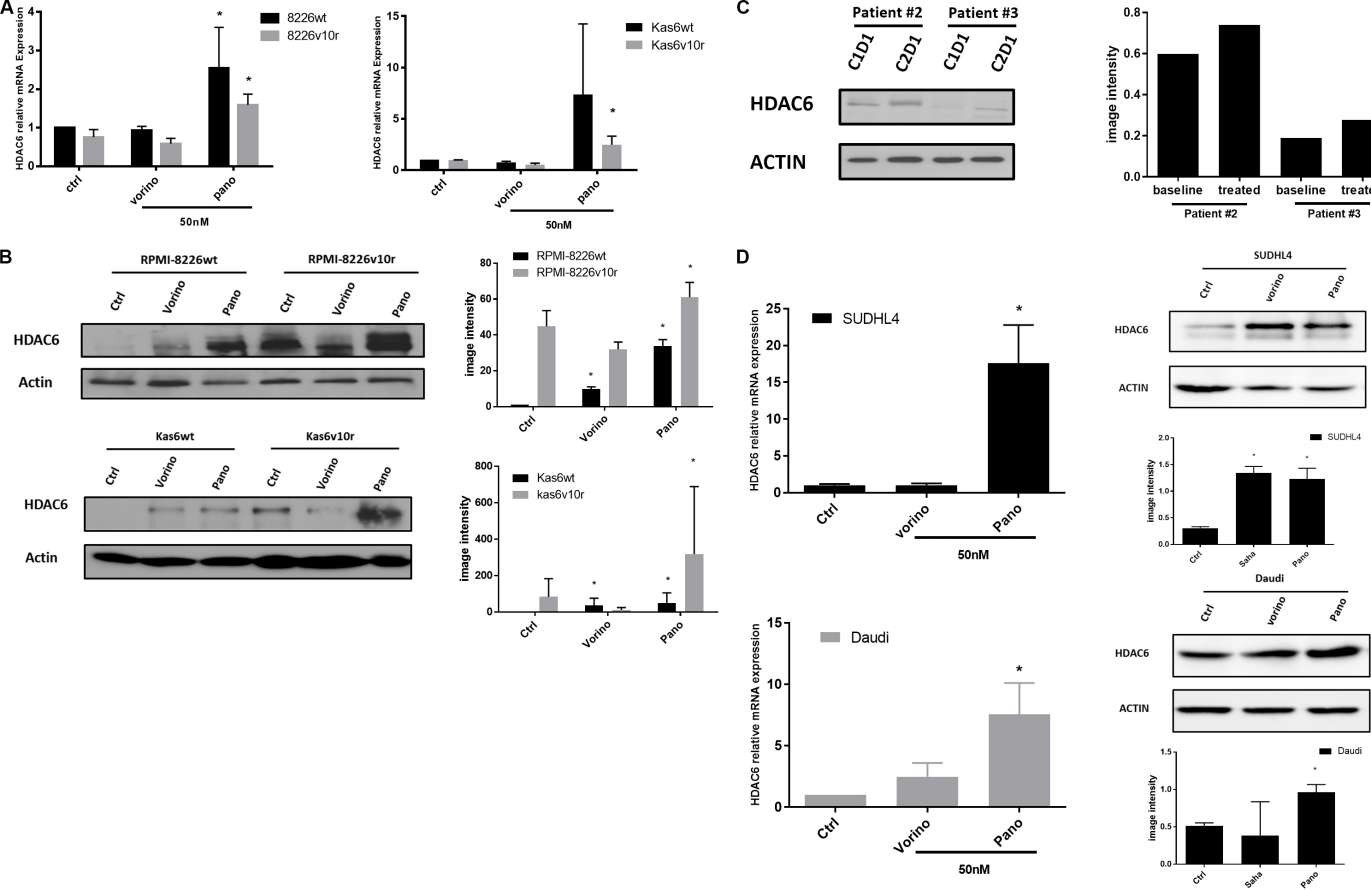
Panobinostat, but not vorinostat, upregulates HDAC6 expression. (A) RMPI-8226 and Kas6 were treated with 50 nM vorinostat or panobinostat for 12 h before harvesting for total RNA, and HDAC6 expression was measured by RT-PCR. Expression of HDAC6 in RMPI-8226 and Kas6 cells treated with vorinostat or panobinostat was normalized to vehicle-treated RPMI-8226wt and Kas6wt, respectively. (B, C) RMPI-8226 and Kas6 were treated with 50 nM vorinostat or panobinostat for 12 h before harvesting for protein, and HDAC6 expression was measured by immunoblotting. Results were quantified by scanning for band density and plotted. Patient sample (peripheral blood) derived from a phase I/II newly diagnosed multiple myeloma clinical trial before and after the first cycle treatment was harvested (Clinicaltrials.gov, number NCT01440582), and HDAC6 expression in PBMC was measured by immunoblotting, and quantitative results were plotted next to immunoblotting. (D) SUDHL4 and Daudi cells were treated with 50 nM vorinostat or panobinostat for 12 h before harvest for total RNA or protein. HDAC6 mRNA expression was measured by RT-PCR. Expression of HDAC6 in cells treated with vorinostat or panobinostat was normalized to vehicle control. HDAC6 expression was measured by immunoblotting. Results were quantified by scanning for band density and plotted. ^*^P < 0.05. HDAC: Histone deacetylase.

Because HDAC6 was increased in both RPMI-8226 and Kas6 resistant lines compared to parental lines [Figure 1], we hypothesized that pan HDACi might modulate the expression of this family member. Therefore we quantified HDAC6 transcript expression before and after vorinostat or panobinostat treatment in wildtype and bortezomib-resistant myeloma cells individually. Panobinostat significantly increased HDAC6 expression, while vorinostat did not alter the expression [Figure 5A] in RPMI-8226wt and RPMI-8226v10r cells alike. Interestingly, panobinostat but not vorinostat also induced substantial HDAC6 expression in both Kas6wt and Kas6v10r [Figure 5A]. Immunoblotting results confirmed the upregulation of HDAC6 protein expression by panobinostat in all four myeloma cell lines [Figure 5B]. Specifically, panobinostat caused HDAC6 to increase by more than 30- and 60-fold in RPMI-8226wt and RPMI-8226v10r cells, respectively, and more than 40- and 300-fold in Kas6wt and Kas6v10r cells, respectively, as quantified by densitometry. The increase of HDAC6 in the cell line models was corroborated by similar HDAC6 upregulation in patient samples treated with panobinostat as described in Figures 4C and 5C. To determine whether this was a myeloma-specific effect, we addressed whether a similar upregulation was observed in lymphoma cells. Indeed, panobinostat increased the expression of HDAC6 [Figure 5D] in both SUDHL4 (a diffuse large B-cell lymphoma cell line) and Daudi (a Burkitt lymphoma cell line), suggesting the HDAC6 upregulation by panobinostat is applicable to both myeloma and lymphoma models.

### Pharmacological targeting of HDAC6 shows synergy with panobinostat in promoting cell death

**Figure 6:**
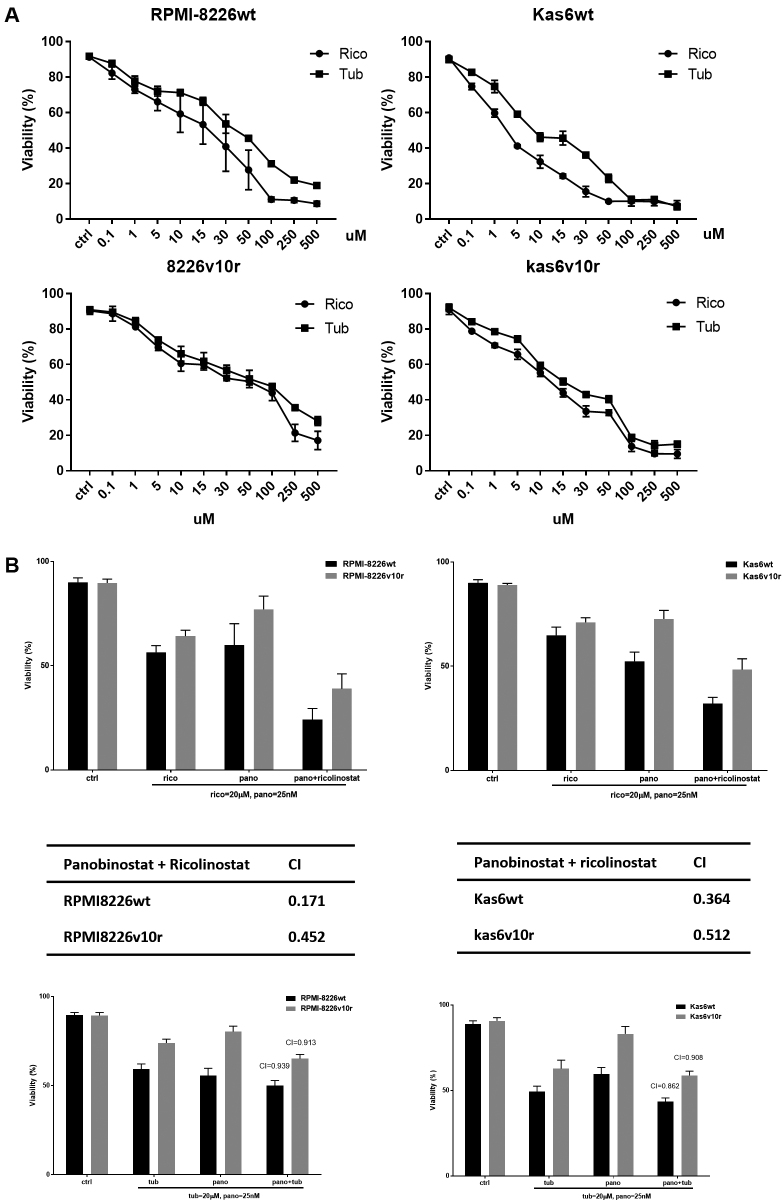
Panobinostat plus pharmacological HDAC6 inhibitors induced cell death synergistically. (A) RMPI-8226 and Kas6 were treated with various concentrations of the HDAC6-specific inhibitors, ricolinostat, or tubaicin for 48 h, and cell viability was measured. Viability was normalized to control treated with vehicle. (B) RPMI8226wt and Kas6wt were treated with single agent ricolinostat, tubaicin or panobinostat; or a combination of ricolinostat plus panobinostat or tubacin plus panobinostat for 48 h. Cell viability was measured after treatment using trypan blue assay, and combination index was calculated by Isobologram analysis. HDAC: Histone deacetylase.

Since HDAC6 transcript and protein expressions were increased after exposure to panobinostat in sensitive and resistant myeloma cells, we hypothesized that HDAC6 specific inhibitors might augment panobinostat efficacy. Parental or bortezomib-resistant myeloma cells were treated with either ricolinostat or tubaicin, two distinct HDAC6-specific pharmacological inhibitors. We found that increasing concentrations of HDAC6 inhibitors decreased the cell viability of both parental and bortezomib-resistant RPMI-8226 and Kas6 cells [Figure 6A]. Adding either of these HDAC6 inhibitors to panobinostat enhanced cell death [Figure 6B], and CI index analysis revealed that ricolinostat or tubaicin plus panobinostat are synergistic in causing a loss of viability, with ricolinostat yielding a lower CI index indicating increased synergy likely due to the higher potency of ricolinostat compared to tubaicin [Figure 6B].

## DISCUSSION

Panobinostat, a potent pan-HDACi, has been approved for refractory multiple myeloma and is the sole HDACi to show significant clinical benefit in this disease^[8]^. However, panobinostat’s documented efficacy is countered by toxicities, specifically gastrointestinal and cardiac issues^[24,25]^. To maximize the efficacy of panobinostat and minimize undesired toxicity, we sought to understand the underlying mechanism of panobinostat from the perspective of targeting selective HDACs. Therefore, we employed isogenic bortezomib-resistant myeloma models to profile the expression of individual HDACs in sensitive versus resistant lines. Surprisingly, we discovered HDAC6 and HDAC7 were upregulated and downregulated, respectively, in two separate models of bortezomib-resistant myeloma cells. Furthermore, we demonstrated that treatment of these lines with panobinostat caused dysregulation of HDACs while vorinostat failed to do so, which suggested an association between HDACi potency and effects on altered expression of HDACs. In particular, panobinostat caused a substantial downregulation of HDAC7 as well as a significant upregulation of HDAC6 in myeloma cells. Similar observations were made in patients enrolled in a clinical trial using panobinostat as first-line therapy, although this conclusion is tempered by the small number of samples analyzed. These data have implications for mechanisms of bortezomib resistance as well as mechanisms of panobinostat activity in the setting of bortezomib resistance, and indicate that further study of these two HDACs may be a key to overcoming refractory multiple myeloma. However, we acknowledge that the underlying mechanism of bortezomib resistance is multifaceted and not limited to IGFR-1 activation^[19]^. Our conclusion that individual HDACs are implicated in bortezomib resistance may not be generalizable because we model bortezomib resistance utilizing activation of IGFR-1 as an underlying mechanism in our cells, and we do not explore other mechanisms of bortezomib resistance. Our data showed that panobinostat decreased HDAC7 transcript and protein expression, and HDAC7 knockdown led to reduced growth and cell cycle redistribution towards a G1 population. Although we did not investigate the downstream targets of HDAC7 that may contribute to the decreased cell growth, HDAC7 regulates cyclin D1 expression in osteogenesis^[31,32]^, and it is possible that this function of HDAC7 is conserved in myeloma. However, it has also been reported that HDAC7 promotes apoptosis in B acute lymphoblastic leukemia^[19]^, which is contradictory to our findings. Therefore, we speculate that HDAC7 function is highly contextual and is dependent upon its presence in nuclei versus cytoplasm^[33]^ and its association with other proteins (including other HDACs such as HDAC3) in transcriptional complexes and the subsequent impact on gene expression^[34]^.

Our data also revealed that panobinostat but not vorinostat upregulated HDAC6 expression in myeloma cells. The *in vitro* data was further supported by the increase of HDAC6 in patient samples treated with panobinostat. HDAC6 is crucial in regulating protein degradation, response to endoplasmic reticular stress, and counteracts apoptosis induced by an overload of unfolded proteins^[35]^. In bortezomib-refractory cells, we found that adding HDAC6 selective inhibitors to panobinostat further increased cell death. More importantly, the CI index analysis revealed that panobinostat synergized with HDAC6 selective inhibitors to enhance cell death. It has been reported that selective HDAC6 inhibitors augmented the anti-tumor activity of pomalidomide^[36]^ as well as bortezomib^[37]^ in myeloma. Our data revealed that HDAC6 inhibition also enhanced panobinostat efficacy, likely due to impaired protein degradation mechanism and increased ER stress. These data have implications for de-escalation of panobinostat when combined with HDAC6 inhibition^[38]^, a strategy that may reduce the toxicities associated with the current dosing of panobinostat.

In summary, our data showed heterogeneous expression of HDACs between bortezomib-sensitive and resistant cells; however, HDAC7 downregulation in resistant lines was striking and consistent across models. A caveat of our study lies in the specific bortezomib refractory lines used. The cells were continuously cultured in bortezomib to maintain resistance. Also, both the resistant lines show increased activation of the IGFR-1, and we did not explore other mechanisms of bortezomib refractory disease to confirm if modulation of HDAC expression was universally conserved. Interestingly, panobinostat caused the further decrease of HDAC7 and increase of HDAC6, which was validated in patients treated with panobinostat. Using CRISPR, we reduced HDAC7 expression and found induction of cell cycle arrest and slowed cell growth. On the contrary, HDAC6 was upregulated by panobinostat, and pharmacological suppression of HDAC6 augmented cell death induced by panobinostat, highlighting an opportunity to combine HDAC inhibitors at low doses. Although we did not elaborate on this observation, our data in Figure 3B shows that panobinostat increases transcript expression of HDAC11 in both bortezomib-sensitive and -refractory cells. A deeper understanding of how overexpression and knockdown of HDAC6, 7, and 11 modulate myeloma survival and drug resistance will enable the development of more precise HDACi strategies. Taken together, these data suggest multifaceted and complex functions of HDACs and HDACi in multiple myeloma and are suggestive of future combination strategies for panobinostat in this disease.

## DECLARATIONS

### Copyright

© The Author(s) 2021.
